# Polymer/BiOBr-Modified Gauze as a Dual-Functional Membrane for Heavy Metal Removal and Photocatalytic Dye Decolorization

**DOI:** 10.3390/polym12092082

**Published:** 2020-09-13

**Authors:** Chi-Jung Chang, Pei-Yao Chao, Chen-Yi Chou, Ying-Jen Chen, Chih-Feng Huang

**Affiliations:** 1 Department of Chemical Engineering, Feng Chia University, 100, Wenhwa Road, Seatwen, Taichung 40724, Taiwan; happinese_cat@yahoo.com.tw (P.-Y.C.); jennychou830404@gmail.com (C.-Y.C.); a7071272@gmail.com (Y.J.C.); 2 Department of Chemical Engineering, i-Center for Advanced Science and Technology (ICAST), National Chung Hsing University, Eng Bld 3, 250 Kuo Kuang Road, Taichung 40227, Taiwan; huangcf@nchu.edu.tw

**Keywords:** adsorbent, photocatalyst, heavy metal, dye, BiOBr, polyethyleneimine

## Abstract

It is crucial to remove heavy metals and dyes before discharging industrial effluents. Gauze substrate was surface-modified by coating with a polymeric adsorbent and a spray coating of BiOBr photocatalyst to develop a novel dual-functional membrane, polymer/BiOBr-modified gauze, for water remediation. The polymeric adsorbent was crosslinked to prevent the dissolving of the adsorbent during operation in contaminated water. The morphology and surface chemistry of the modified gauze were characterized before and after the adsorption of Ni^2+^. The surface wettability, isotherms, and kinetics of Ni^2+^ adsorption were studied. We also studied the effect of pH, initial Ni^2+^ concentration, monomer molar ratio, and monomer chemical structure on the Ni^2+^ adsorption capacity. To achieve a high Ni^2+^ adsorption capacity and good photocatalytic decolorization activity, the amount of decorated BiOBr was tuned by changing the spray-coating time to optimize the exposed BiOBr and polymer on the surface. The optimized dual-functional membrane PB20 possesses excellent adsorption capacity (650 mg g^−1^) for Ni^2+^ ions and photocatalytic decolorization activity (100% degradation of RhB within 7 min). Decorating the optimized amount of BiOBr on the surface can introduce photocatalytic decolorization activity without sacrificing the adsorption capacity for Ni^2+^.

## 1. Introduction

Heavy metal ions in industrial effluents have been a serious problem. Soluble heavy metal ions will accumulate in living organisms, leading to some diseases. Several heavy metals are found in the effluents of many industries. Among them, nickel is a transition metal with moderate toxicity. It can cause skin disorders, such as allergic reactions and dermatitis. Additionally, chronic exposure may result in some problems, such as pulmonary fibrosis and cardiovascular diseases [1,2,3]. Therefore, toxic heavy metal ions should be removed from industrial effluents to minimize the environmental impact of effluents toward water bodies. Furthermore, colorants (dye and pigment) are widely utilized in textile, paper, leather, polymer, cosmetics, food, and chemical industries [4,5]. There has been an urgent need to remove heavy metals and dyes in industrial wastewater by appropriate processes [6,7,8,9,10,11]. 

At present, various techniques, such as ion-exchange [12,13], chemical precipitation [14,15], photocatalytic degradation [16,17,18], Fenton process [19], membrane separation [20,21,22], and adsorption [23,24,25], etc., have been developed for the removal of single pollutants (either heavy metal or dye). Among them, adsorption is still an effective method because it is efficient, low cost, and easy to handle. With the use of dyes and heavy metals in various industries, dyes and heavy metals were reported to coexist in industrial effluents [26,27]. Both heavy metals and dyes have significant ecological effects on our environment due to their toxicity, persistence, and bioaccumulation. It is very important to remove heavy metals and dyes before discharging industrial effluents [28]. Effective removal of both hazardous dyes and potentially toxic metals is not easy work due to the different physical and chemical properties of dyes and heavy metals, such as the treatment of MO and Pb(II) [29]. A great deal of effort has been devoted to the development of effective, facile, and environmentally friendly approaches for the simultaneous removal of heavy metals and dyes from contaminated water. Skold et al. reported the simultaneous metal and organic (perchloroethylene, PCE) complexation by (carboxymethyl)-β-cyclodextrin [30]. Wan et al. [31] reported that manganese-oxidizing bacteria consortia exhibited effective removal of Mn(II) in harsh conditions. Manganese-oxidizing bacteria and biogenic Mn oxides are useful in the remediation of pollution containing organic pollutants and heavy metal ions. 

Nowadays, there are some studies about the removal of heavy metals and dyes from wastewater [32,33,34,35]. Inorganic nanomaterial/polymer composites have attracted much attention due to the increasing demand for new materials with improved photocatalytic, adhesion, mechanical, photoconductive, adhesion, and thermal properties [36,37,38,39,40,41,42,43,44]. Liu et al. reported that silver nanoparticles and polypyrrole-modified BiOBr with a Z-scheme heterostructure acted as a composite catalyst that possesses outstanding photocatalytic activity and stability [45]. Additionally, polyethyleneimine (PEI) has excellent metal chelation abilities because of the large amount of −NH_2_ groups on its molecular chain [46,47]. Wang et al. [48] found that polyethyleneimine-functionalized ion-imprinted hydrogel was useful for the removal of Cu (II).

In this study, we attempted to remove dyes and heavy metals by adsorption and photocatalysis reactions, respectively, using inorganic nanomaterial/polymer composites (polymer/BiOBr-modified gauze). We used a crosslinked polymer consisting of hydrophilic diepoxy (PEGDE), a crosslinking agent (PEI), and diamine (TETA) to obtain a polymer-modified gauze, and then functionalized with BiOBr through spray coating to prepare the polymer/BiOBr-modified gauze as a dual functional membrane that possesses a high adsorption capacity for Ni^2+^ ion and good photocatalytic decolorization activity. The morphology and surface chemistry of the modified gauze were characterized before and after the adsorption of Ni^2+^. The surface wettability, isotherms, and kinetics of adsorption were studied. We also studied the effect of pH, initial Ni^2+^ concentration, monomer molar ratio, and monomer chemical structure on the Ni^2+^ adsorption capacity. To achieve a high Ni^2+^ adsorption capacity and good photocatalytic decolorization activity, the amount of decorated BiOBr was tuned by changing the spray-coating time to optimize the exposed BiOBr and polymer on the surface.

## 2. Materials and Methods 

Gauze substrate (100% Rayon) was coated with an aqueous solution of hydrophilic monomers, including diepoxy (poly(ethylene glycol) diglycidyl ether, (PEGDE)), diamine (triethylenetetramine, (TETA)), and a crosslinking agent (polyethyleneimine (PEI)). However, since all monomers are well soluble in water, the coated samples were thermally cured to fix the polymeric adsorbent on the gauze by an amine-epoxy crosslinking reaction to prevent the dissolving of adsorbent during operation in contaminated water. Then, BiOBr was dispersed in the above-mentioned solution and deposited on the polymer-modified gauze by spray-coating and thermal curing to obtain the polymer/BiOBr-modified gauze as a dual functional membrane that possesses high adsorption capacity for Ni^2+^ ion and good photocatalytic decolorization activity.

### 2.1. Materials

Poly(ethylene glycol) diglycidyl ether (PEGDE), and triethylenetetramine (TETA) were purchased from Aldrich (St. Louis, MO, USA). Polyethylenimine (PEI) was supplied by Alfa-Aesar (Tewksbury, MA, USA). 1,4-bis(3-aminopropyl)-piperazine (BAP) was materials purchased from TCI (Fukaya City, Japan).

### 2.2. Preparation of BiOBr

BiOBr was prepared by a solvothermal method. First, 0.12 g of bismuth nitrate was added to the mixed solution containing 5 mL of ethylene glycol and 35 mL of isopropanol. Then, 0.18 g of cetyltrimethylammonium bromide (CTAB) was added. The solution was stirred for 60 min and then transferred to a Teflon-lined vessel. The reaction was carried out at 145 °C for 12 h. After being washed in deionized water and ethanol solution, filtration, and drying, bismuth bromide powder (BiOBr) can be obtained.

### 2.3. Coating of Immobilized Adsorbents on gauze@PEI0.4/PEGDE1/TETA0.6

Polyethylenimine (PEI, 0.4 mmole), poly(ethylene glycol) diglycidyl ether (PEGDE, 1 mmole), and triethylenetetramine (TETA, 0.6 mmole) are all dissolved in DI water to prepare a solution with a solid content of 10 wt%. A 4 cm × 4 cm gauze is dip-coated in the above-mentioned solution, then thermal cured at 115 °C for an hour. The polymer-modified gauze is designated as gauze@PEI_x_/PEGDE_y_/TETA_z_ where x, y, and z mean the mole feed ratio of PEI, PEDGE, and TETA monomers, respectively. 

### 2.4. Preparation of Polymer/BiOBr-modified Gauze

**Scheme 1:**
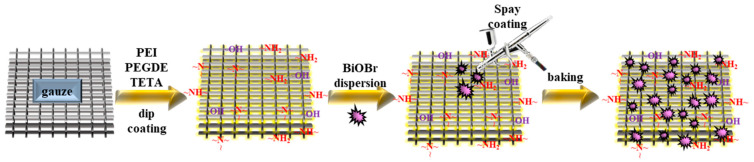
Process for the preparation of polymer/BiOBr-modified gauze.

The prepared BiOBr suspension (5 wt% in ethanol) was loaded in an airbrush (Sparmax DH-103) with a 0.3-mm nozzle. Then, the suspension was spray-coated on the surface of the gauze@ adsorbent substrates (4 cm × 4 cm) layer by layer. Three samples (PB10, PB20, PB30) were prepared by changing the time for spray coating (10, 20, 30 s). The samples were denoted as PBX. P means the polymer-modified gauze sample (gauze@PEI_0.4_/PEGDE_1_/TETA_0.6_). B indicates BiOBr photocatalyst. X indicates the time for spray coating. The process for the preparation of polymer/BiOBr-modified gauze (Section 2.3 and Section 2.4) is demonstrated in Scheme 1. 

### 2.5. Characterization of Modified Gauze

A field emission scanning electron microscope (HITACH S-4800) was used to observe the surface textures and elemental analysis for samples. The conditions for mapping include acceleration voltage 15 kV, working distance 8 mm. XPS (ULVAC-PHI, PHI 5000 Versa Probe) was used to investigate the surface chemistry of the samples. The Ni^2+^ contents in the solution were monitored by an ultraviolet-visible spectrophotometer (JASCO, V-770). The dynamic contact angles of the samples were monitored by the contact angle meter (CAM-100).

### 2.6. Batch Adsorption Study

The modified gauze (4 × 4 cm) was immersed in an aqueous solution containing Ni^2+^ (1600 mg/L) at room temperature for 90 min. An aliquot of the solution was withdrawn from the reaction mixture. The residual Ni^2+^ content in the solution was determined by the UV–VIS spectrometer. 

The capacity of Ni^2+^ adsorption was obtained from the equation [49]:

Adsorption capacity

(1) $$\left(\mathrm{Qe}\right)=\frac{\left(C_{0}-C_{e}\right)V}{m}\left(\mathrm{mg}/g\right)$$

where m and V are the weight of adsorbent (g) and the volume of solution (L), respectively. C_e_ and C_o_ are the final and initial ion concentrations (mg/L). 

The adsorption kinetics was investigated by pseudo first or second-order models. The appropriateness of different models was determined by the correlation coefficient (R^2^). The model is more suitable if the R^2^ value is closer to 1. The equations for first order and second order are listed below [50]:

log(Qe − Qt ) = log Qe − k_1_t/2.303 (2)

1/(Qe − Qt) = 1/Qe + k_2_t (3)

The adsorption between the residual and adsorbed metal ions in the solution during the adsorption process can be estimated by the adsorption isotherms using Langmuir and Freundlich models [51]. In this study, equilibrium isotherms are measured to evaluate the capacity of the adsorbent for the adsorption of Ni^2+^.

Langmuir adsorption assumes that the largest adsorption is related to a saturated surface monolayer of solute molecules on the adsorbent. The equation is listed as follows:

1/qe = (1/qm kL)1/Ce + 1/qm(4)

where Ce, qm, qe, and kL represent the equilibrium concentration of metal ions (mg/L), monolayer adsorption capacity (mg/g), quantity of adsorbed metal ion (mg/g), and equilibrium Langmuir constant (L/mg), respectively. 

At equilibrium, the amount of adsorbed adsorbate per unit mass of the adsorbent (qe) can be investigated by the Freundlich adsorption isotherm [52]:

log qe =log kF + 1/nlog Ce(5)

where n and kF mean the values determined graphically, and Freundlich constants, respectively.

### 2.7. Photocatalytic Activity

Before the photocatalytic degradation test, the modified gauze (4 cm × 4 cm) was immersed in the solution and stirred in the dark for 60 min to reach an adsorption-desorption equilibrium between the modified gauze and RhB dye. When the photocatalytic reaction began, 10 mL of aqueous RhB solution (10 ppm) was stirred and kept at 30 °C when irradiated by a Xe lamp. The change in concentration of RhB was monitored every 15 min by the UV–VIS spectrometer. The decolorization rate can be obtained as:

Decolorization (%) = C/C_0_ × 100 (%) (6)

where C_0_ and C are the initial dye concentration and the dye concentration after light irradiation, respectively. The absorption peak intensity of RhB at 554 nm was measured to monitor its decolorization rate.

## 3. Results and Discussion

### 3.1. The Removal of Heavy Metal Ions

#### 3.1.1. Dynamic Contact Angle (DCA)

**Figure 1:**
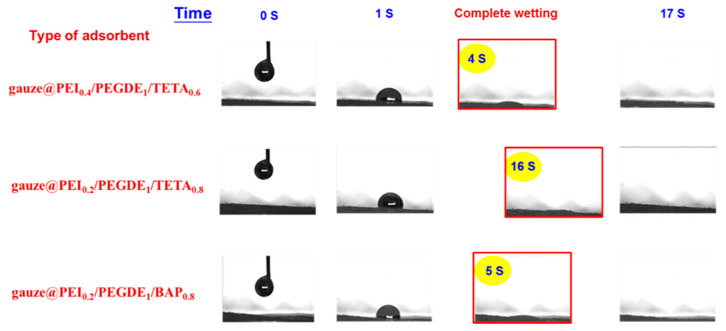
Images of the time required for the complete wetting of water droplet on various polymer-coated gauze.

Figure 1 shows the images of the dynamic contact angle test, revealing the time required for the complete wetting of water droplets on various polymer-coated gauze. All samples are hydrophilic. The water droplet penetrated the adsorbents (complete wetting) within a short period of time. The complete wetting time for different membranes gauze@PEI_0.4_/PEGDE_1_/TETA_0.6_, gauze@PEI_0.2_/PEGDE_1_/TETA_0.8_, and gauze@PEI_0.2_/PEGDE_1_/BAP_0.8_ is 4, 16, and 5 s, respectively. 

However, the result highlighted that the dynamic wetting behavior for each adsorbent is different. For example, the time needed for complete wetting on gauze@PEI_0.2_/PEGDE_1_/TETA_0.8_ (16 s) is longer than that of gauze@PEI_0.4_/PEGDE_1_/TETA_0.6_, which only takes 4 s. The longer time taken by gauze@PEI_0.2_/PEGDE_1_/TETA_0.8_ is ascribed to its lower mole ratio of PEI monomer than that in gauze@PEI_0.4_/PEGDE_1_/TETA_0.6_. Furthermore, the dynamic contact angle also evidenced the effect of different types of amino monomer on the wettability of the adsorbents. Although both adsorbents have the same mole ratio of PEI, when TETA is replaced by BAP, the time required for complete wetting decreases significantly from the initial 16 s (gauze@PEI_0.2_/PEGDE_1_/TETA_0.8_) to 5 s (gauze@PEI_0.2_/PEGDE_1_/BAP_0.8_). 

#### 3.1.2. Charaterization of the Gauze@PEI_0.4_/PEGDE_1_/TETA_0.6_ (FESEM and Mapping)

**Figure 2:**
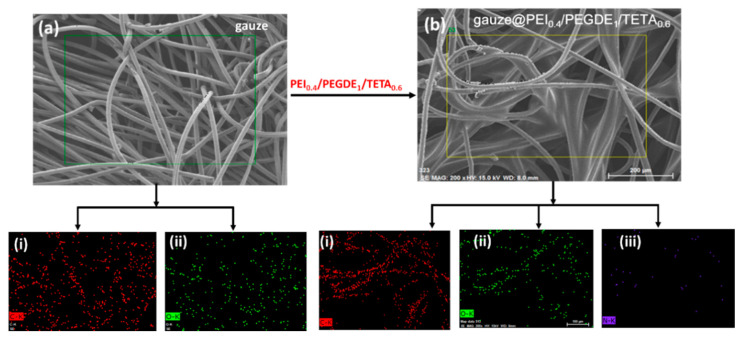
FESEM images of (**a**) pristine gauze and (**b**) gauze@PEI_0.4_/PEGDE_1_/TETA_0.6_. Elemental C, O, and N mapping of the pristine gauze.

**Figure 3:**
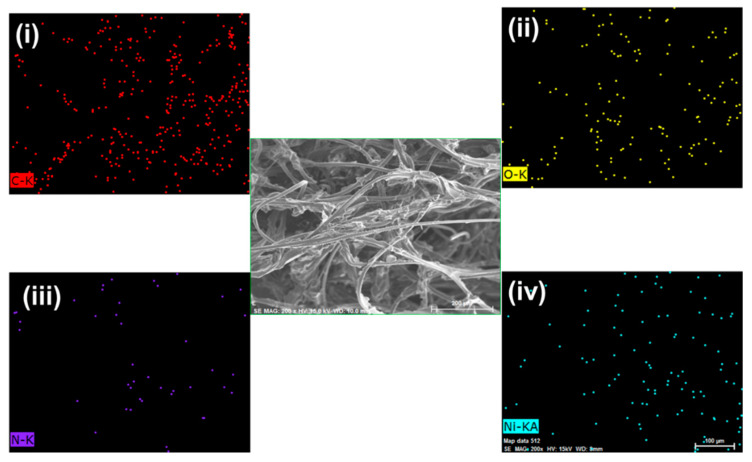
SEM image of Ni^2+^ adsorbed gauze@PEI_0.4_/PEGDE_1_/TETA_0.6_, and (**i**) C, (**ii**) O, (**iii**) N, and (**iv**) Ni^2+^ mapping.

Figure 2 presents the SEM images of gauzes and its elemental distribution before and after adsorption. It is observed that there is a significant difference between pristine gauze (Figure 2a) and gauze@PEI_0.4_/PEGDE_1_/TETA_0.6_ (Figure 2b). Pristine gauze consists of stacks of fibers, thus leading to the presence of some interstitial voids. The SEM image of gauze@PEI_0.4_/PEGDE_1_/TETA_0.6_ displayed that the presence of the PEI_0.4_/PEGDE_1_/TETA_0.6_ coating leads to an increase in the diameter of the coated fiber as well as fill up the interstitial voids. Additionally, the uniform surface of the gauze revealed that the adhesion between the coated polymeric adsorbent layer and gauze is good. The mapping imaging of N (Figure 2b (iii)) showed that the amine functional group of the polymeric adsorbent was uniformly distributed on the gauze. Figure 3 shows the SEM images and elemental mapping of gauze@PEI_0.4_/PEGDE_1_/TETA_0.6_. After the adsorption of Ni^2+^, the surface of the gauze became rough. The adsorption of Ni^2+^ significantly changed the surface morphology of the sample. Further, the results of elemental mapping confirmed the adsorption of nickel on the adsorbent layer of polymer-modified gauze.

#### 3.1.3. XPS Spectra of Gauze@PEI_0.4_/PEGDE_1_/TETA_0.6_

**Figure 4:**
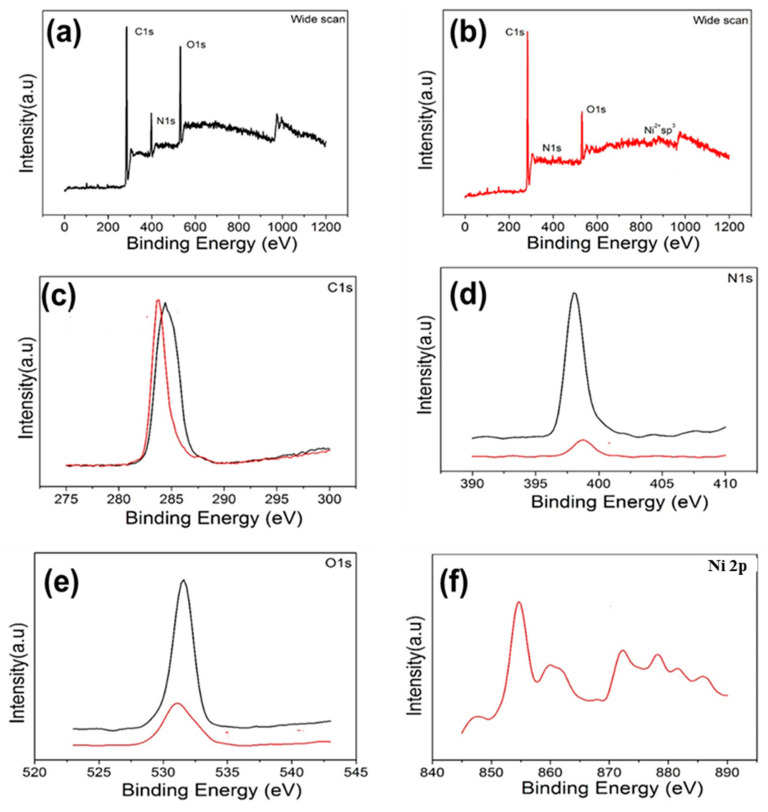
Wide-scan XPS spectra of (**a**) gauze@PEI_0.4_/PEGDE_1_/TETA_0.6_ and (**b**) Ni^2+^ adsorbed gauze@PEI_0.4_/PEGDE_1_/TETA_0.6_. XPS spectra of (**c**) C 1s; (**d**) N 1s; (**e**) O 1s, and (**f**) Ni 2p regions in gauze@PEI_0.4_/PEGDE_1_/TETA_0.6_ (black line) and gauze@PEI_0.4_/PEGDE_1_/TETA_0.6_ after Ni^2+^ adsorption (red line).

The chemical compositions of the polymer-modified sample, gauze@PEI_0.4_/PEGDE_1_/TETA_0.6_ are monitored by XPS. The wide-scan XPS spectra of pristine and Ni^2+^ adsorbed gauze@PEI_0.4_/PEGDE_1_/TETA_0.6_ are illustrated in Figure 4a,b, respectively. The C, O, and N elements were found in the XPS spectra of both gauzes, indicating the coating of polymeric adsorbent on the gauze. However, Ni is detected for the Ni^2+^-adsorbed sample (Figure 4b). Figure 4c–f indicates the XPS spectra of elements C, N, and O, respectively. The C 1s peak at 283.8 eV (Figure 4c) and a N 1s peak at 398 eV (Figure 4d) indicated the presence of cross-linked polymeric adsorbent containing PEI and TETA. However, the characteristic peak of N 1s is shifted towards larger binding energy of 398.7 eV in the Ni^2+^-adsorbed sample (Figure 4d), confirming the successful chelation between Ni^2+^ ions and the amine group of PEI and TETA segments on the polymer chain. Figure 4e exhibits the binding energy around 531.5 eV for O 1s that is associated with the oxygen element in C-OH in the samples. According to Figure 4e, the binding energy of O 1s shifted towards 531 eV for the gauze@PEI_0.4_/PEGDE_1_/TETA_0.6_ after Ni^2+^ adsorption. This can be attributed to the decrease in electron density around oxygen atoms as they lose electrons that migrated to Ni^2+^ after the chelation between OH and Ni^2+^ [53]. The shift shows that, due to Ni^2+^ adsorption, the oxygen atom tends to exist in a more oxidized state. As shown in Figure 4f, various Ni species can be interpreted in the spectrum of Ni 2p with a prominent peak at around ~854.1 eV for Ni(0) [54]. Additionally, it is also observed that Ni^2+^ is reduced to Ni(0) because of the coordination between Ni^2+^ and amine groups in TETA and PEI. The energy spectrum of the Ni 2p peak is shown clearly in Figure 4f. It shows a major peak at 857 eV, indicating the presence of oxidized Ni. Furthermore, there are a few more minor peaks observed at 862.1 eV, 874.2 eV, 879.1 eV, 881.2, and 889 eV, which are attributed to the presence of various Ni oxide species, such as Ni(OH)_2_ and NiOOH [55]. Thus, the results revealed that Ni^2+^ acquired electrons and formed a complex between Ni and NH_2_.

#### 3.1.4. Effect of Solution pH toward the Adsorption Process

**Figure 5:**
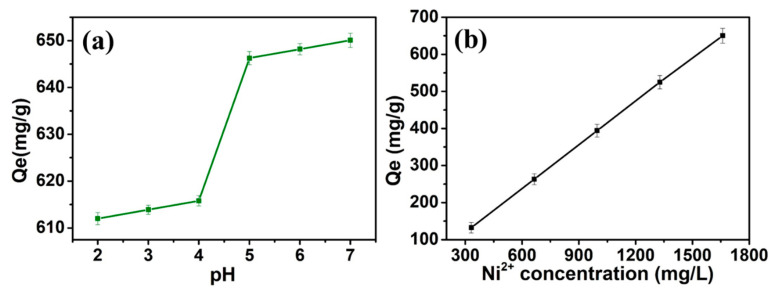
Effect of (**a**) pH (**b**) initial Ni^2+^ concentration on the adsorption capacity of gauze@PEI_0.4_/PEGDE_1_/TETA_0.6_.

The effects of the pH of the solution on Ni^2+^ adsorption and desorption performance by polymeric adsorbent-coated gauze were investigated. As shown in Figure 5a, the influence of pH on the Ni^2+^ adsorption is determined by a solution with various pH (from 2 to 7). Our result demonstrated that the adsorption capacity of Ni^2+^ varies with increasing pH value. The lower Ni^2+^ adsorption capacity at low pH (pH 2–4) is ascribed to the larger positive charge density of the polymeric adsorbent due to the protonation reaction (H^+^ + NH_2_ → NH_3_^+^), thus increasing the repulsion between Ni^2+^ ions. Additionally, the lower pH of the solution leads to great competition among metal ions with an abundance of hydrogen ions. Similar results were also reported by Chang and coworkers [56]. For acidic solutions, the transformation of -NH_2_ into NH^3+^ causes a few -NH_2_ sites on the surface of the adsorbents that can react with heavy metal ions. With the increase in pH value, the adsorption of Ni^2+^ also increases. The maximum uptake of Ni^2+^ ions is achieved at pH 7. This results from the deprotonation of more functional groups that are accessible to heavy metal ions at higher pH values when compared to lower pH. A further increase in pH leads to the formation of precipitates. Hence, the remaining experiments in this study are carried out at pH = 7.0.

#### 3.1.5. Effect of Initial Concentration on Ni^2+^ Adsorption

The effect of the initial concentration of Ni^2+^ towards the adsorption capacity of Ni^2+^ is investigated using various concentrations ranging from 300 mg/L to 1600 mg/L. The investigation is performed at pH 7.0 for 90 min to ensure equilibrium is achieved. Figure 5b clearly shows that the adsorption capacity of Ni^2+^ can be enhanced with increasing initial concentration of Ni^2+^. The result is attributed to the enhanced chelation between the adsorbent and Ni^2+^ ions. Moreover, the increase in the concentration of heavy metal ions will lead to enhanced ion adsorption. A similar phenomenon was reported Kesenci et al. [57].

#### 3.1.6. Adsorption Isotherms

**Figure 6:**
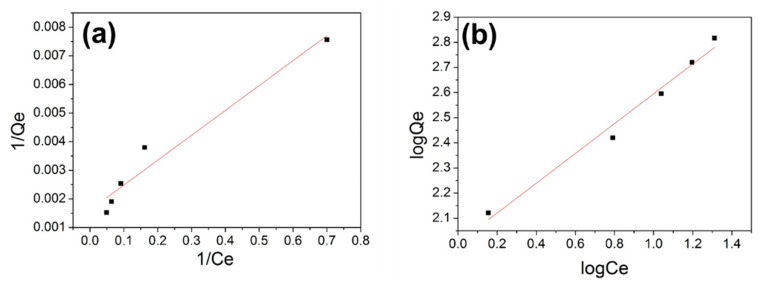
Adsorption isotherms of gauze@PEI_0.4_/PEGDE_1_/TETA_0.6_ based on (**a**) the Langmuir isotherm model and (**b**) the Freundlich isotherm model.

According to both experimental results for the adsorption of Ni^2+^ versus equilibrium concentration. Results were analyzed by the Freundlich and Langmuir equations at different temperatures [58]. Linear plots were obtained for both cases, revealing the Freundlich and Langmuir isotherms can be applied to the Ni^2+^ adsorption of this study. Langmuir and Freundlich plots for the Ni^2+^ adsorption are shown in Figure 6a,b, respectively. The derived Langmuir and Freundlich constants are listed in Table 1. In comparison with the Langmuir model, the Freundlich model demonstrated a better fit. It is worth noting that the correlation coefficient (R^2^) for gauze@PEI_0.4_/PEGDE_1_/TETA_0.6_ presented in the Freundlich isotherms is closer to 1 when compared to the Langmuir isotherms. Thus, the adsorption process of Ni^2+^ tends to occur on the heterogeneous surface.

#### 3.1.7. Adsorption Kinetics

**Figure 7:**
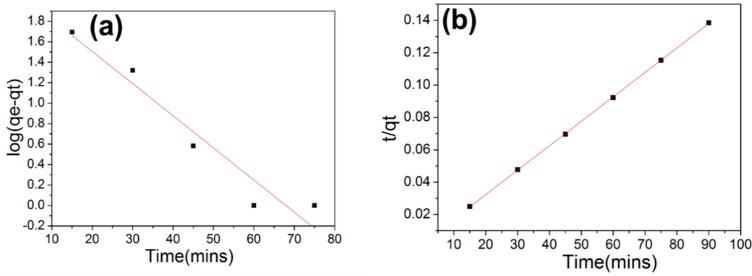
Adsorption kinetics of Ni^2+^ ions on gauze@PEI_0.4_/PEGDE_1_/TETA_0.6_ fitted in (**a**) pseudo first-order model and (**b**) pseudo second-order model.

The reactions and mechanism of the adsorption process can be investigated based on the adsorption kinetic data. Further, the adsorption kinetic data also provides the information on the adsorption of solutes at a solid-liquid interface which, in turn, is influenced by the residence time of Ni^2+^. The kinetic data were fitted in two types of the kinetic model including pseudo first-order and second-order models, as shown in Figure 7. Table 2 lists the results of the linear fitting. Hence, based on the correlation coefficient, the adsorption of Ni^2+^ on modified gauze is best described using a pseudo second-order model.

#### 3.1.8. Effect of the Types and the Molar Ratio of Amine Monomers on Ni^2+^ Removal

**Figure 8:**
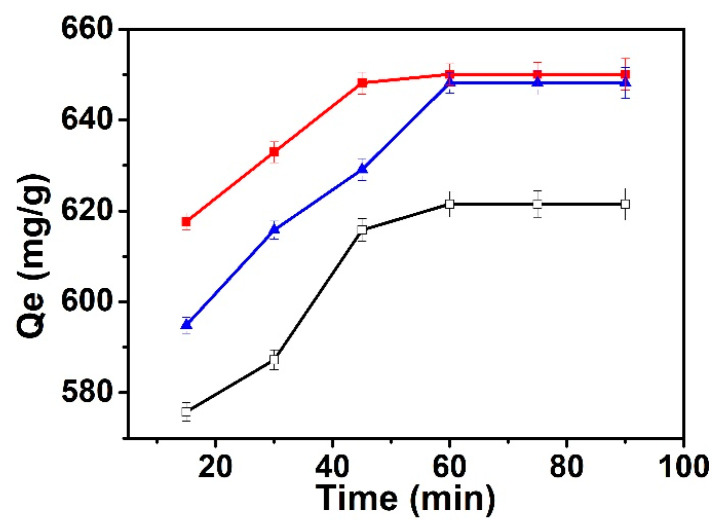
Adsorption capacity towards Ni^2+^ of gauze@PEI_0.4_/PEGDE_1_/TETA_0.6_ (■); gauze@PEI_0.2_/PEGDE_1_/TETA_0.8_ (□) and gauze@PEI_0.2_/PEGDE_1_/BAP_0.8_ (▲).

To check the influence of the structure of adsorbent on the adsorption capacity, a part of the PEI monomer is replaced by linear (TETA) or cyclic (BAP) amino monomers. The relationship between the molar ratio of both PEI/TETA and PEI/BAP with the adsorption efficiency is presented in Figure 8. The sample with a higher PEI content (PEI_0.4_/PEGDE_1_/TETA_0.6_) demonstrated a higher adsorption capacity of Ni^2+^ than that of immobilized adsorbents with a lower PEI content (PEI_0.2_/PEGDE_1_/TETA_0.8_). The better adsorption capacity of PEI_0.4_/PEGDE_1_/TETA_0.6_ is ascribed to the higher content of amino groups available on the polymer chain of PEI_0.4_/PEGDE_1_/TETA_0.6_ in comparison with that of PEI_0.2_/PEGDE_1_/TETA_0.8_. It is also reported in the research done by Chen et al. [59] where the adsorption capacity of TET-MRGO is enhanced after the incorporation of TETA when compared to pristine GO. The higher adsorption efficiency of triethylenetetramine magnetic-reduced graphene oxide composite (TET-MRGO) is due to the higher amount of NH_2_- groups, which also acts as the binding sites for Cu(II) ions. Therefore, with the loading of TETA, the stability and adsorption capability of graphene oxide is greatly enhanced. The results proved that the composition (chemical structure) of the polymeric adsorbent not only affects the surface wettability, but also the absorption performance.

Additionally, as seen in Figure 8, the adsorption capacity of PEI_0.2_/PEGDE_1_/BAP_0.8_ towards Ni^2+^ is significantly higher than that of PEI_0.2_/PEGDE_1_/TETA_0.8_. Hence, we can deduce that the immobilized adsorbents containing BAP amino groups are more preferably to undergo chelation with Ni^2+^ ions which in turn enhancing the removal of Ni^2+^ ions. Researchers found the ligand-to-metal charge-transfer (LMCT) caused by a transfer of electrons from the tertiary amine to the metal center. Yamamoto et al. observed the UV–VIS absorbance increases in poly(amidoamine) (PAMAM) dendrimers chelation of Pt^2+^, attributing them to LMCT [60]. This phenomenon can be interpreted as a transfer of electrons from the HOMO of the tertiary amine to the metal center LUMO, causing the compound to adapt its unique color. Mankbadi and coworkers [61] reported the chelation of iron ions by generation 4 hydroxyl-terminated PAMAM. It is a LMCT between the free electron group of the dendrimer’s internal amines and the dehalogenated Fe^3+^ ion. In this study, there are two primary amine and two cyclic tertiary amine groups in a BAP monomer. There are two primary amine and two linear secondary amine groups in a TETA monomer. The results observed in this study reveals that BAP amino groups more preferably to undergo chelation with Ni^2+^ ions than TETA groups. The cyclic tertiary amine groups favor the ligand-to-metal charge-transfer.

Surprisingly, as seen in Figure 8, gauze@PEI_0.4_/PEGDE_1_/TETA_0.6_ exhibited the best adsorption capacity among all three immobilized adsorbents. Hence gauze@PEI_0.4_/PEGDE_1_/TETA_0.6_ is chosen to be used throughout the rest of the study. Nevertheless, the adsorption capacity of all three immobilized adsorbents increases with time and remains constant after 80 min as seen in Figure 8. Thus, the reaction time for the rest of the experiments was carried out in a constant amount of time, 80 min. It is worth noting that equilibrium can be reached within such a short period of contact time, suggesting that the adsorbents show an excellent affinity towards Ni^2+^ ions. 

### 3.2. Polymer/BiOBr-modified Gauzes as Dual-Functional Membranes

#### 3.2.1. Morphology

**Figure 9:**
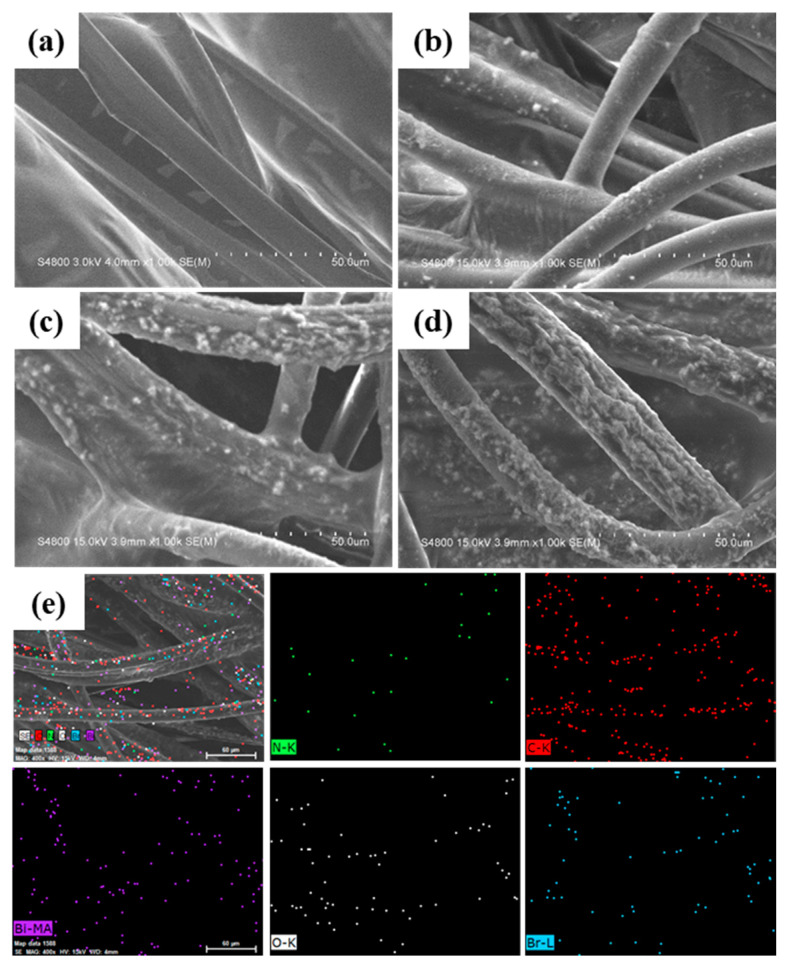
FESEM images of polymer-modified gauze (**a**) P, and polymer/BiOBr-modified gauze samples prepared by changing the time for spray coating (10, 20, 30 s) (**b**) PB10, (**c**) PB20, (**d**) PB30, and (**e**) elemental N, C, Bi, O, Br mapping images of PB30.

FESEM was used to study the morphology of polymer-modified gauze and polymer/BiOBr-modified gauze samples (Figure 9). In the case of polymer-modified gauze (Figure 9a), the fibers exhibit smooth surfaces. In addition to the coating on the fiber surface, some cavities among fibers were filled by the polymeric adsorbent. In comparison with the polymer modified gauze, polymer/BiOBr-modified gauze sample PB10 (Figure 9b) shows some BiOBr nanomaterials on the surface of some fibers. The presence of BiOBr in PB10 can be observed based on the light-colored areas. As the spraying time increases, the PB20 sample (Figure 9c) has more BiOBr nanomaterials on the surface than the PB10 sample. These BiOBr were well-dispersed on the surface with minor agglomeration. For the PB30 sample (Figure 9d), the higher amount of BiOBr causes agglomeration of the particles on the fiber surface. The surface turned from smooth to rough as the spraying time increase. Figure 9e presents the N, C, Bi, O, Br elemental mapping images of PB30. The elemental mapping imaging of nitrogen (N) proved that the uniform distribution of the amine functional group of the polymeric adsorbent on the gauze. Additionally, the elemental mapping of Bi, O, and Br confirmed the decoration of BiOBr on the surface of the polymer-coated gauze.

#### 3.2.2. Dynamic Contact Angle (DCA)

**Figure 10:**
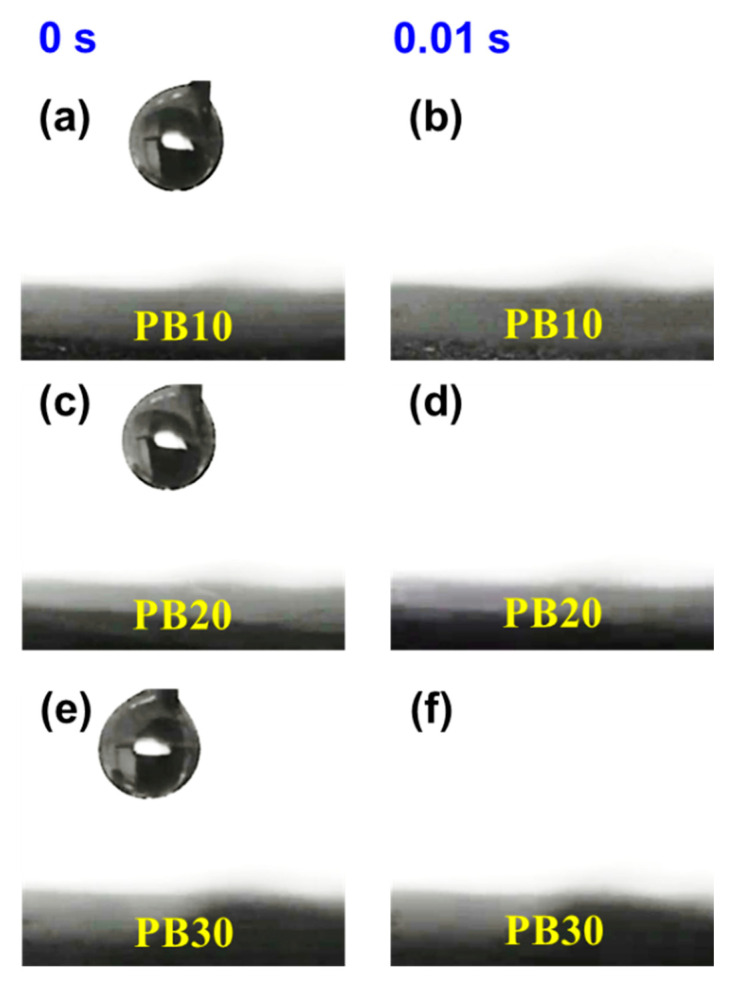
Initial images of (**a**) PB10 (**c**) PB20 (**e**) PB30 and dynamic contact angle images of the time required for the complete wetting of water droplets on various polymer/BiOBr-coated gauze (**b**) PB10 (**d**) PB20 (**f**) PB30.

As shown in Figure 1, the complete wetting time for the polymer-modified gauze membrane gauze@PEI_0.4_/PEGDE_1_/TETA_0.6_ is 4 s. However, the decoration of BiOBr makes the membranes even more hydrophilic. Figure 10 presents the initial images of (a) PB10 (c) PB20 (e) PB30 and dynamic contact angle images of the time required for the complete wetting of water droplets on various polymer/BiOBr-coated gauze (b) PB10 (d) PB20 (f) PB30. All three polymer/BiOBr-modified gauze membranes with different amounts of BiOBr can be completely wetted within 0.01 s.

#### 3.2.3. Photocatalytic Decolorization Performance

**Figure 11:**
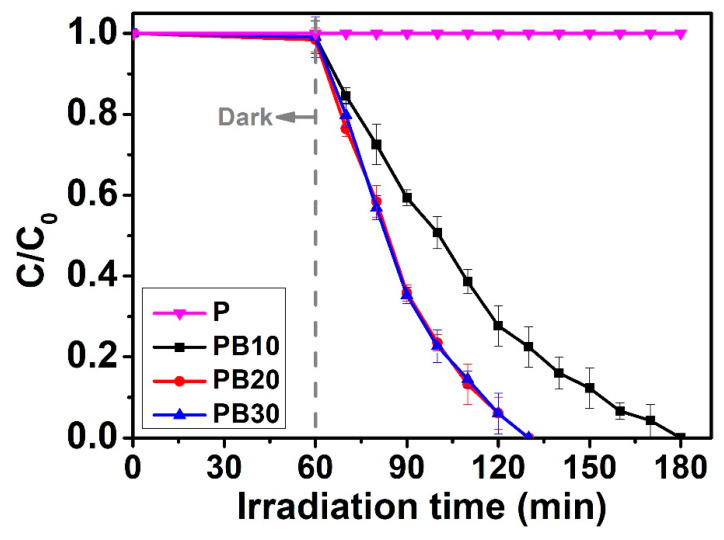
Photodegradation curves of RhB over polymer-modified gauze (P), and polymer/BiOBr-modified gauze samples (PB10, PB20, PB30).

The RhB dye was selected to survey the photocatalytic activity of the polymer-modified gauze, and polymer/BiOBr-modified gauze samples (PB10, PB20, PB30). Photodegradation curves are demonstrated in Figure 11. The control experiment was performed without adding any BiOBr photocatalyst on polymer-modified gauze (P) to realize the decolorization nature of P under the irradiation of light. From the blank experiment, it is revealed that RhB was not decolorized by P under the light irradiation. The decoration of BiOBr on the adsorbent layer introduces the photocatalytic activity. It takes 120 min for 100% degradation of RhB by PB10. PB20 and PB30 showed excellent photocatalytic activity with 100% degradation of RhB over 70 min than that of PB10 (78% degradation). PB30 with more BiOBr did not show better activity than PB20 with fewer BiOBr. As demonstrated in Figure 9d, agglomeration of the BiOBr particles was found on the fiber surface. The effective surface active area of BiOBr cannot increase because of the agglomeration. This may be the reason why PB20 and PB30 showed similar photocatalytic activity.

#### 3.2.4. Effect of BiOBr Decorization on Ni^2+^ Removal

**Figure 12:**
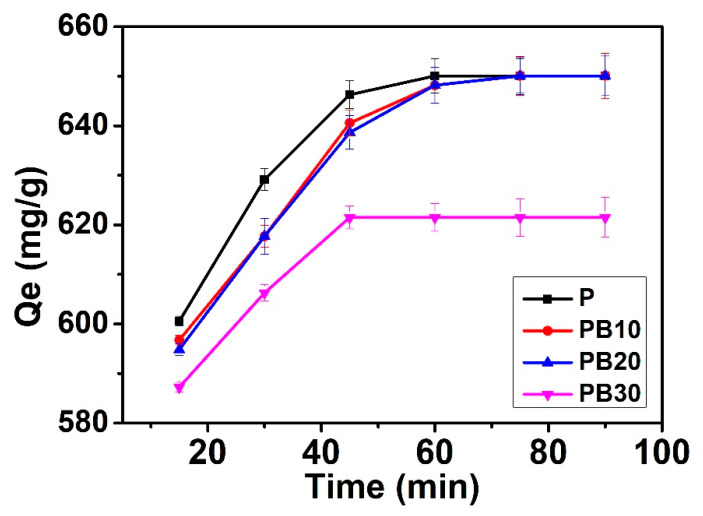
Adsorption capacity towards Ni^2+^ of polymer-modified gauze (P), and polymer/BiOBr-modified gauze samples (PB10, PB20, PB30).

Adsorption capacity towards Ni^2+^ of polymer-modified gauze (P), and polymer/BiOBr-modified gauze samples (PB10, PB20, PB30) are shown in Figure 12. The control experiment was performed without adding any BiOBr photocatalyst on polymer-modified gauze (P) to realize the Ni^2+^ adsorption capacity of P. From the blank experiment, it is revealed that P possesses excellent adsorption capacity of 650 mg g^−1^ for Ni^2+^ ions. After the decoration of BiOBr on the adsorbent layer, the area of exposed polymeric adsorbent (PEI_0.4_/PEGDE_1_/TETA_0.6_) decreased. The results in Figure 12 showed that PB10 and PB20 samples had similar Ni^2+^ adsorption capacity (650 mg g^−1^) as P. However, the Ni^2+^ adsorption capacity of PB30 decreased to 620 mg g^−1^. The decreased area of exposed polymeric adsorbent may be the reason for the decreased adsorption capacity. Considering the compromise between photocatalytic activity and Ni^2+^ adsorption capacity, PB20 is the optimized dual-functional polymer/BiOBr-modified gauze membrane.

## 4. Conclusions

In this study, polymer/BiOBr-modified gauzes were developed as dual-functional membranes for the removal of organic dyes and heavy metal ions. The gauze substrate was surface-modified by coating with a polymeric adsorbent consisting of hydrophilic diepoxy (PEGDE), a crosslinking agent (PEI), and diamine (TETA) to develop a novel dual-functional photocatalyst and adsorbent membrane for water remediation. Since all monomers are well soluble in water, the coating was crosslinked to prevent the dissolving of the adsorbent on the polymer-modified gauze during operation in contaminated water. For the gauze@PEI_0.4_/PEGDE_1_/TETA_0.6_ membrane, the adsorption isotherms were better described by the Freundlich model. The XPS results revealed that Ni^2+^ acquired electrons and formed a complex between Ni and NH_2_ during the Ni^2+^ adsorption process. The maximum uptake of Ni^2+^ ions is achieved at pH 7. The adsorption capacity of Ni^2+^ increases with the increase in the initial concentration of Ni^2+^. The monomer molar ratio and monomer chemical structure each show a great influence on the Ni^2+^ adsorption capacity. Then, BiOBr was dispersed in ethanol and deposited on the polymer-modified gauze by spray-coating to obtain the polymer/BiOBr-modified gauze. To achieve a high Ni^2+^ adsorption capacity and good photocatalytic decolorization activity, the amount of decorated BiOBr was tuned by changing the spray-coating time to optimize the exposed BiOBr and polymer on the surface. The amount of BiOBr on the surface increased with increasing the spray coating time. The optimized dual-functional membrane PB20 possesses an excellent adsorption capacity (650 mg g^−1^) for Ni^2+^ ions and photocatalytic decolorization activity (100% degradation of RhB within 70 min). Decorating the optimized amount of BiOBr on the surface can introduce photocatalytic decolorization activity without sacrificing the adsorption capacity for Ni^2+^. Polymer/BiOBr-modified gauze has great potentials in the remediation of wastewater containing organic pollutants and heavy metals.

## Figures and Tables

**Table 1:** Freundlich and Langmuir isotherm constants.

Adsorbent	Langmuir Isotherm			Freundlich Isotherm		
	Q_m_ (mg/g)	K_L_ (L/mg)	R^2^	K_F_	n	R^2^
gauze@PEI_0.5_/PEGDE_1_/TETA_0.6_	625	5.43	0.9454	100.4	1.7	0.982

**Table 2:** Parameters used for the kinetic model.

Adsorbent	Pseudo First-Order			Pseudo Second-Order		
	q_e_ (mg/g)	K_1_ (h^−1^)	R^2^	q_e_ (mg/g)	K_2_ (g/h mg)	R^2^
gauze@ PEI_0.4_/PEGDE_1_/TETA_0.6_	126	0.005	0.916	666	0.001	0.999
